# Biomimetic lung-targeting vehicle coupled with cryo-shocked leukocyte and inflammation-anchored liposome for drug delivery and anti-inflammation in treatment of acute pneumonia

**DOI:** 10.1093/rb/rbag032

**Published:** 2026-03-10

**Authors:** Jinniu Zhang, Yixing Zeng, Yun Huang, Chunyun Zhu, Wenhao Shen, Yingjing Miao, Nianping Feng, Tianyuan Ci

**Affiliations:** School of Pharmacy, Shanghai University of Traditional Chinese Medicine, Shanghai 201203, China; School of Pharmacy, Shanghai University of Traditional Chinese Medicine, Shanghai 201203, China; School of Pharmacy, Shanghai University of Traditional Chinese Medicine, Shanghai 201203, China; School of Pharmacy, Shanghai University of Traditional Chinese Medicine, Shanghai 201203, China; School of Pharmacy, Shanghai University of Traditional Chinese Medicine, Shanghai 201203, China; School of Pharmacy, Shanghai University of Traditional Chinese Medicine, Shanghai 201203, China; School of Pharmacy, Shanghai University of Traditional Chinese Medicine, Shanghai 201203, China; School of Pharmacy, Shanghai University of Traditional Chinese Medicine, Shanghai 201203, China

**Keywords:** cryo-shocking, leukocytes, targeting drug delivery, inflammation targeting, pneumonia

## Abstract

Acute pneumonia is a severe pulmonary inflammation, and it is critical to promptly suppress the dysregulated inflammatory responses to prevent mortality. Glucocorticoids are the first-line therapeutic drugs but with poor tissue selectivity and dose-dependent adverse effects. In this work, cryo-leukocyte, an autologous cell-derived immunosuppressor, was created by leveraging the cryo-shocking technology by the quick shock of normal leukocytes with liquid nitrogen. After coupling with aICAM-1 functionalized liposomes, this micro/nano composite system could achieve efficient and prompt inflammation alleviation in acute pneumonia. The engineered cryo-leukocytes were of well biocompatibility after evaluation of blood toxicity, tissue toxicity, acute toxicity and long-term biosafety for over 6 months, etc. Cryo-leukocytes preserved similar cellular receptors as normal leukocytes, capable of recognizing and binding inflammatory cytokines but without activation of immune cascade, thus exhibiting obvious anti-inflammation efficacy by acting as ‘mixed cytokines antibodies’. The immunosuppression efficacy of cryo-leukocytes was also superior than that of its sub-group cells of cryo-neutrophil, cryo-monocyte and cryo-lymphocyte, due to relative wide protein expressions that are related to the immune responses. Besides, cryo-leukocytes coupled with aICAM-1 functionalized liposome exhibited obvious anchoring effect in inflammation sites by the interaction of ICAM-1 antibody and ICAM-1 molecules that were over-expressed on inflammatory pulmonary endothelial cells, thus served as superior drug lung-targeting vehicle to maximally enhance the accumulation of traditional Chinese and Western medicines in the lungs. A total of 68.1% of drug signals could be observed in lung tissues compared with other major organs after intravenous injection, significantly higher than that of micro-sized drug-loaded cryo-leukocyte (18.6%) and nano-sized drug-loaded aICAM-1-liposome (12.2%). In a lipopolysaccharide-induced acute pneumonia mice model, the drug-loaded cryo-leukocyte achieved superior anti-inflammation efficacy with 87.5% survival of mice after treatment.

## Graphical Abstract

**Graphical Abstract:**
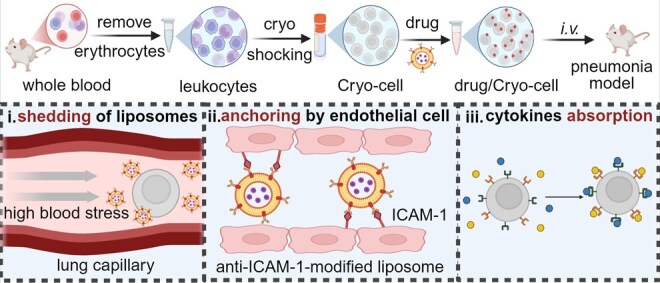

## Introduction 

Acute pneumonia is a severe inflammation disorder of respiratory system, characterized by hyperactivation of the immune system and excessive release of pro-inflammatory cytokines [1]. This dysregulated immune response often leads to hypoxemia, acute respiratory failure or even multi-organ dysfunction syndrome [2, 3]. The mortality rate associated with acute pneumonia is as high as 50% and timely control of uncontrolled inflammation is important to improve clinical outcomes [4, 5].

Glucocorticoids remain the first-line therapeutic intervention for acute pneumonia management [6, 7]. However, their systemic distribution with poor tissue selectivity and dose-dependent adverse effects (excessive immunosuppression, osteoporosis, osteonecrosis, etc.) have raised substantial clinical concerns [8]. This limitation underscores the needs to develop advanced therapeutic biologics with enhanced efficacy and optimized safety profiles.

Lung-targeted drug delivery systems have been extensively employed in the treatment of pulmonary diseases [9, 10]. The particle size of the carrier is a critical factor influencing lung targeting. Directly selecting micrometer-scale vehicles represents a straightforward approach to achieve pulmonary drug targeting *via* capillary entrapment [11]. However, traditional polymeric micrometer-scale carriers, such as microspheres, often exhibit high material rigidity and low deformability, which may cause vascular embolism and impair respiratory function. In contrast, nanoscale drug carriers like liposomes and nanoparticles face challenges of the low lung-anchoring efficiency, and a significant portion tends to accumulate in the liver other than lung tissues [12].

Leukocytes are critical components of the immune system [13]. Rather than representing a single cellular entity, they encompass diverse immunocompetent cells including granulocytes (neutrophils, eosinophils, basophils), monocytes and lymphocytes (T cells, B cells, NK cells) [14]. These immune cells play vital roles in mediating the progression of severe inflammation [15–17]. Our previous work has proposed a cryo-shocking technology [18], which enables preservation of cellular proteins and receptors while terminating cell proliferation, and we herein adapt this platform for leukocyte engineering to create cryo-leukocyte, an autologous cell-derived immunosuppressor, leveraging its capability to recognize and capture proinflammatory cytokines.

Besides, cryo-leukocytes underwent transient trapping in lung capillaries due to their cellular size and functioned as a natural drug lung-targeting material. Meanwhile, leveraging the pathological environment of pneumonia that ICAM-1 receptor is over-expressed on vascular endothelium [19, 20], the nano-sized ICAM-1 antibody (aICAM-1) modified liposomes were integrated in delivery vehicle to facilitate the anchoring and long retention of drugs in lungs.

**Figure 1:**
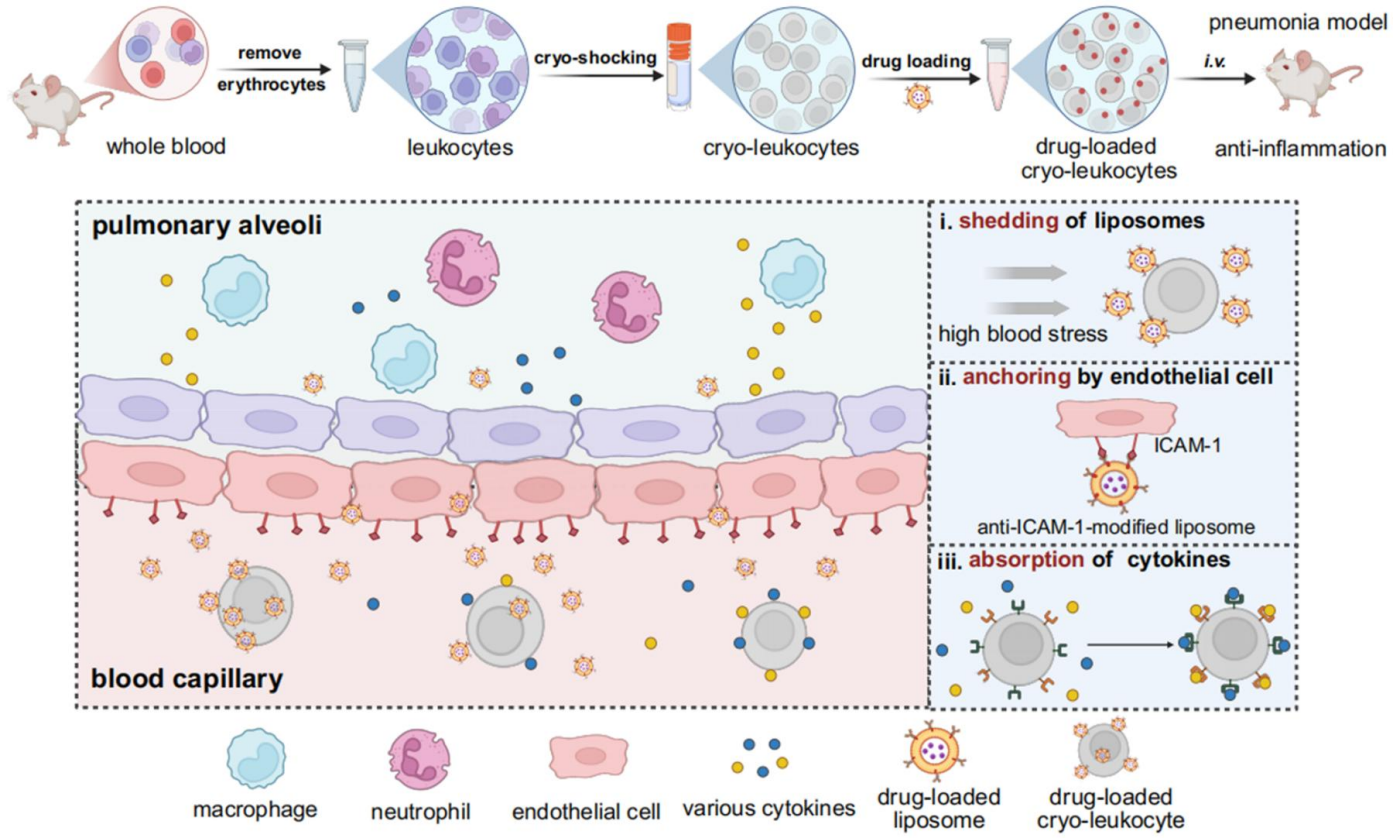
Preparation scheme of the drug-loaded cryo-leukocytes and the *in vivo* anti-inflammation mechanisms. Created with BioRender (www.biorender.com).

In specific, in this work we encapsulated the two anti-inflammation drugs of methylprednisolone (MP) and baicalin (BAI) in aICAM-1 modified cationic liposome (drug@Lip), and further loaded it in cryo-leukocyte by electrostatic interaction (drug@Lip/cryo-leukocyte). Under high shearing stress encountered in microvasculature, the attached liposomes dissociated from cryo-leukocyte and underwent ICAM-1-mediated endothelial targeting and anchoring. Afterwards, cryo-leukocyte operated as a cytokine-capturing material, synergizing with the delivered anti-inflammatory drugs to ameliorate inflammation of acute pneumonia (Figure 1).

## Materials and methods

### Materials

Methylprednisolone, baicalin, lecithin, cholesterol, octadecylamine were bought from Aladdin Corporation (Shanghai, China). Lipopolysaccharide (LPS) was provided by Shanghai Yuanye Biotechnology Co., Ltd (Shanghai, China). The enzyme-linked immunosorbent assay (ELISA) kits (IL-6, TNF-α, IL-1β, CXCL2) and fluorescence-labeled antibodies (APC-CXCR2, APC-TLR4, PE-CD121a, PE-gp130, PE-CD120a, PE-CD14, PE-F4/80, APC-CD11b, PE-Ly6G) were purchased from Biolegend Corporation (CA, USA). Rhodamine-labeled phalloidin (Rhodamine-Phalloidin) was purchased from Suzhou UElandy Biotechnology Co., Ltd (Suzhou, China). Hoechst 33342, Dulbecco’s Modified Eagle Medium (DMEM) and anhydrous Dimethyl sulfoxide (DMSO) were provided by Jiangsu Kaiji Biotechnology Co., Ltd (Jiangsu, China). Calcein/PI cell viability kit was purchased from Beyotime Biotechnology Co., Ltd (Shanghai, China). Fetal bovine serum (FBS) and cell counting kit-8 (CCK-8) were provided by Dalian Meilun Biotechnology Co., Ltd (Dalian, China). C57BL/6J female mice were supplied by Animal Experimental Center of Shanghai University of Traditional Chinese Medicine. All animal tests complied with the animal protocol were approved by the institutional animal care and use committee of Shanghai University of Traditional Chinese Medicine (PZSHUTCM2310300004).

### Preparation of cryo-leukocyte

The whole blood was first collected from the orbital vein of mice and centrifuged at 450 *g* for 5 min to obtain the cell pellets. The cells were treated with ammonium chloride-potassium (ACK) buffer for 3 min to lyse the erythrocytes and centrifuged at 450 *g* for 5 min after adding 10-fold of phosphate-buffered saline (PBS), yielding leukocyte pellets in the bottom. After washing with PBS, the leukocytes were resuspended in a medium composed of 70% DMEM, 20% FBS and 10% DMSO. The cell-containing medium was then quickly immersed in liquid nitrogen for at least 12 h. Prior to use, cryo-leukocytes were thawed at 37°C and washed with PBS twice.

### Characterization of cryo-leukocyte

The cellular structure of cryo-leukocyte was analyzed using confocal laser scanning microscopy (CLSM) and scanning electron microscopy (SEM). For CLSM analysis, the cells were suspended in 200 μL PBS and stained with 5 μL rhodamine-phalloidin solution at room temperature for 25 min. Subsequently, the cells were centrifuged at 450 *g* for 5 min and washed with PBS. The resulting pellets were then resuspended in 500 μL Hoechst 33342 staining solution and incubated for another 15 min. The stained cryo-leukocytes were thoroughly washed and resuspended in PBS prior to CLSM (SP-8, Leica) observation. To assess cell viability, the cryo-leukocytes were stained with calcein AM and propidium iodide (PI) and observed by CLSM. For SEM characterization, both cryo-leukocytes and normal leukocytes were fixed with 3.5% glutaraldehyde solution at 4°C for 4 h. The cells were then treated with 1% osmium tetroxide for 1 h. Following fixation, the cells were subjected to gradient dehydration with increasing concentrations of ethanol. Subsequently, the cells were treated with a 1:1 (v/v) mixture of ethanol and isoamyl acetate for 30 min and then suspended in pure isoamyl acetate overnight. Finally, the prepared samples were deposited onto silica plates. After drying and sputter-coating with gold, the samples were imaged with SEM (SU8010, HITACHI).

The *in vitro* proliferation of cryo-leukocyte was further analyzed with the CCK-8 assay. Briefly, both cryo-leukocytes and normal leukocytes were suspended in cell culture medium and seeded into a 96-well plate at a cell density of 5 × 10^3^ per well. After culturing for 0.5, 24, 48 and 72 h, 10 μL of CCK-8 solution was added to each well, and the cells were incubated for an additional 3 h. The absorbance of each sample was measured at 450 nm using a microplate reader (Spark 10M, TECAN).

### Cytokine secretion of cryo-leukocyte

The cryo-leukocytes were seeded in a 24-well plate at a cell density of 1 × 10^6^ per well and stimulated with LPS at a concentration of 50 ng/mL. In contrast, normal leukocytes stimulated with LPS served as the positive control, while cryo-leukocyte without LPS stimulation were used as the negative control. After 6 h, the supernatant of the cell culture medium was collected and centrifuged at 300 *g* for 10 min. The levels of cytokines IL-6, TNF-α and IL-1β were measured by ELISA kits.

### Biosafety evaluation of cryo-leukocyte

The blood biocompatibility of cryo-leukocyte was first evaluated. In brief, cryo-leukocytes were suspended in 5 mL of PBS to prepare samples with varying cell densities (1 × 10^5^, 1 × 10^6^, 5 × 10^6^, 1 × 10^7^ cells/mL). PBS and deionized water were served as the negative and positive control, respectively. Blood was collected from the orbital vein of the mice into the anticoagulation tubes (heparin sodium as the anticoagulant), and 100 μL of fresh blood was added to above cell suspensions. The mixture was then equilibrated in a 37°C water bath for 1 h and centrifuged at 200 *g* for 5 min. The supernatants were collected, and the absorbance of each sample was measured at 540 nm using a microplate reader.

For long-term biosafety evaluation, saline (control group) and cryo-leukocytes (5 × 10^6^ cells per mouse) were intravenously administered in healthy C57BL/6J mice, and the survival and body weight of mice were monitored for 6 months. Additionally, on the 30th day post-injection, the mice in a separate animal batch were euthanized and primary organs (heart, liver, spleen, lung and kidney) were collected for hematoxylin and eosin (H&E) staining to assess tissue microstructure and inflammation status.

For acute toxicity analysis, different doses of cryo-leukocytes (1 × 10^5^, 1 × 10^6^, 5 × 10^6^, 1 × 10^7^, 5 × 10^7^ and 1 × 10^8^) were injected intravenously into healthy mice for 14 consecutive days to observe the toxic symptoms and recorded the mortality and body weight of mice. On day 14, the mice of a separate batch were sacrificed, and typical organs of liver, heart, spleen, lung and kidney were taken out. After H&E staining, the organs were observed under a microscope and photographed.

To evaluate the *in vivo* degradation of cryo-leukocytes, cells were labeled with NHS-cy5.5 to obtain the fluorescence-tagged cryo-leukocyte (cy5.5@cryo-leukocyte). After intravenous injection into mice, the fluorescence intensities over time were monitored using an IVIS imaging system, and the percentage of the retention of cryo-leukocytes was calculated relative to the initial fluorescence intensity on day 0 (set as 100%).

The biosafety of cryo-leukocyte was also evaluated in a LPS-induced pneumonia model [21, 22]. LPS at a dose of 10 mg/kg was instilled into the trachea *via* endotracheal intubation to establish a pneumonia model. Four hours later, the model mice were intravenously injected with saline, cryo-leukocyte (5 × 10^6^ cells) or normal leukocytes (5 × 10^6^ cells), respectively. The survival of the mice was monitored and recorded.

### Protein expression of cryo-leukocyte

To evaluate the protein expression of cryo-leukocytes, the whole-cell protein expression was analyzed using sodium dodecyl sulfate–polyacrylamide gel electrophoresis (SDS-PAGE). Proteins were extracted using RIPA lysis buffer supplemented with a protease inhibitor cocktail. Protein concentrations in each group were quantified using the bicinchoninic acid (BCA) assay. The loading samples were denatured at 95°C for 15 min and electrophoresed in 10% tris-glycine gels at 80 V with 20 µg of protein loaded per well for 3.5 h. After staining with Coomassie brilliant blue and washing with distilled water, the gels were imaged using a digital camera. Protein retention on the surface of cryo-leukocyte was assessed using immunofluorescence staining. In addition, cryo-leukocytes were suspended in 500 μL of PBS containing 2% FBS and stained with fluorescence-labeled antibodies (APC-CXCR2, APC-TLR4, PE-CD121a, PE-gp130, PE-CD120a and PE-CD14). After incubation for 30 min, 10 mL of PBS containing 2% FBS was added, and the samples were centrifuged at 450 *g* for 5 min twice. Subsequently, 300 μL of PBS containing 2% FBS was added to resuspend the cells, and the fluorescent signal was detected using CLSM and flow cytometry (CytoFLEX, Beckman). Normal leukocytes were treated with the same procedures as the control group.

### Cytokine absorption by cryo-leukocyte

IL-6 was labeled with FAM-NHS and incubated with cryo-leukocytes for 30 min. After washing with PBS, the un-attached cytokines were removed by centrifugation. The washed cryo-leukocytes were resuspended in 500 μL of Hoechst 33342, incubated for 30 min in the dark and observed using CLSM.

### *In vitro* immunosuppression of cryo-leukocyte

The mononuclear macrophage cell line J774A.1 was seeded into a 24-well plate at a density of 1 × 10^6^ cells per well. After 12 h, LPS (50 ng/mL) was added to each well to activate the cells for 2 h. Subsequently, different groups of saline and cryo-leukocyte (1 × 10^5^ cells per well) were added to each well and incubated for another 2, 6, 10 and 22 h. The cell supernatant was collected after centrifugation at 300 *g* for 10 min at the indicated time points, and the levels of cytokines (IL-6, TNF-*α*, IL-1β and CXCL2) secreted by J774A.1 cells were measured using ELISA kits. It is generally considered that mouse CXCL2 are functional homologues cytokine of human IL-8 with its receptor of CXCR2 in mice [23, 24]. Thus, in this work we evaluate the secretion of CXCL2 of mice instead.

### Preparation of the sub-population of cryo-leukocyte

Peripheral blood was collected and diluted with an equal volume of PBS. Then the cells were sub-grouped by density gradient centrifugation with Percoll reagent. In brief, 62% and 25% Percoll solutions were gently layered into a centrifuge tube from bottom to top sequentially. The diluted blood sample was slowly added into the tube (a sample ratio of 0.5 mL per 10 mL of gradient material) and centrifuged at 400 *g* for 20 min at 25°C. The separated neutrophils, monocytes and lymphocytes were then cryo-shocked by liquid nitrogen following the same protocol used for cryo-leukocyte to obtain cryo-neutrophil, cryo-monocyte and cryo-lymphocyte.

### *In vitro* anti-inflammation efficacy of different cryo-cells

J774A.1 cells were seeded in a 24-well plate at a density of 1 × 10^6^ cells per well. After 12 h, LPS at 50 ng/mL was added to each well to activate the cells for 2 h. Subsequently, different groups of saline, cryo-leukocyte, cryo-neutrophil, cryo-monocyte and cryo-lymphocyte (1 × 10^5^ cells per well) were added to each well and incubated for another 6 h. The cell supernatant was collected after centrifugation at 300 *g* for 10 min at indicated time points, and the levels of cytokines secreted by J774A.1 cells were measured using ELISA kits.

### *In vivo* anti-inflammation efficacy of different cryo-leukocyte

LPS at a dose of 10 mg/kg was instilled into the trachea *via* endotracheal intubation to set up the pneumonia model. Four hours later, cryo-leukocyte (5 × 10^6^ cells), cryo-monocyte (5 × 10^6^ cells), cryo-lymphocyte (5 × 10^6^ cells), cryo-neutrophil (5 × 10^6^ cells), dexamethasone (DEX, 2 mg/kg) and MP (6 mg/kg) were administered intravenously, respectively. Twenty-four hours after LPS instillation, peripheral blood was collected from the orbital vein into the serum collection tubes and the supernatant was collected after centrifugation at 800 g for 10 min, and cytokine levels in the serum were measured with ELISA kits. Meanwhile, the survival of mice was recorded.

### Proteomic analysis of different cryo-cells

The cryo-cells of cryo-leukocyte, cryo-neutrophil, cryo-monocyte and cryo-lymphocyte were washed with PBS and resuspended to a cell density of 1 × 10^7^ per vial. The proteins in cryo-cells were extracted and underwent alkylation and digestion to obtain the peptides fractions. For each sample, 200 ng of peptides were analyzed with high-performance liquid chromatography–tandem mass spectrometry (HPLC–MS/MS, nanoElute2, Bruker, Germany) and the data were processed with the software of Spectronaut 19.0. Differentially expressed proteins (DEPs) were defined as fold change >1.5, in which up-regulated proteins of fold change >4, down-regulated proteins of fold change <0.25. Venn analysis, volcano analysis, hierarchical clustering analysis and gene ontology annotation (GO) enrichment analysis were conducted by R programming language.

### Preparation of drug-loaded cryo-leukocyte

The drug-loading process consisted of two main steps, the preparation of drug-loaded aICAM-1-modified liposomes (drug@Lip) and their subsequent incubation with cryo-leukocytes. In brief, drug-loaded liposomes were prepared using the film hydration method. Specifically, a mixture of 51.5 mg lecithin, 14.7 mg cholesterol, 5.15 mg MP, 5.15 mg BAI, 3.5 mg octadecylamine and 0.4 mg DSPE-PEG-Streptavidin were added to a round-bottom flask and dissolved in 14 mL of a chloroform–methanol solvent mixture (3:4, v/v). The sample was evaporated under vacuum using a rotary evaporator (25°C) until a uniform film was formed. Next, 6 mL of PBS containing biotinylated ICAM-1 antibody was added to the film for hydration. The solution was then subjected to ultrasonication for 20 min (40 Hz probe) and filtered through 0.45 and 0.22 μm membranes. The liposomes were further ultrafiltrated (Mw 300 kD) to remove the un-linked antibodies. The modification amount of anti-ICAM-1 was detected by BCA assay (Thermo Fisher). The resulting drug@Lip was further incubated with cryo-leukocytes for 0.5 h, and the drugs were loaded *via* electrostatic interactions between cationic liposomes and cryo-leukocytes. After centrifugation at 450 *g* for 5 min, drug@Lip/cryo-leukocyte was obtained.

### Characterization of drug@Lip/cryo-leukocyte

For drug-loading analysis, the loaded drugs were extracted from the preparation using 2 mL of acetonitrile and subjected to ultrasonication for 15 min (40 Hz probe). After centrifugation at 14000 g for 10 min, the supernatant was analyzed by high-performance liquid chromatography (HPLC, 1260 Infinity II, Agilent) to determine the drug concentration. To visualize the drug-loading state in cryo-leukocyte, coumarin 6 (Cou-6) was used as the model drug with the same preparation procedure. The fluorescent signal of the sample was observed by CLSM.

### *In vitro* drug release of drug@Lip/cryo-leukocyte

Drug release from cryo-leukocyte was evaluated using a peristaltic pump to simulate the *in vivo* blood flow conditions. Briefly, Cou-6 was used as the model drug, and the initial fluorescence intensity of Cou-6@Lip/cryo-leukocyte was measured and recorded. The shearing forces of 0, 2 and 6 Pa were adjusted by the flow rate of peristaltic pump. The fluorescence intensity of circulating medium was measured at 10, 30, 60 and 90 min in separated samples after centrifugating the medium at 450 *g* for 5 min. The percentage of drug release at each time point was calculated relative to the initial fluorescence intensity, which was set as 100%.

### Lung targeting of drug@Lip/cryo-leukocyte

Free cy5.5 was used as the model drug and encapsulated in anti-ICAM-1 modified liposomes to prepare cy5.5@Lip. The positively charged cy5.5@Lip was then incubated with cryo-leukocyte to obtain cy5.5@Lip/cryo-leukocyte. Besides above two groups, cryo-leukocytes were modified with NHS-cy5.5 to yield fluorescence-tagged cryo-leukocyte. The fluorescence-tagged cryo-leukocyte was prepared *via* the chemical reactions between the amino groups on cells and N-hydroxysuccinimide (NHS) group on fluorescence molecules. In brief, cy5.5-NHS solution was incubated with the prepared cryo-leukocyte suspension, with the cell concentration of 1 × 10^7^ cells/mL and cy5.5-NHS concentration of 80 μg/mL. After 1 h, the cell suspension was centrifuged to remove the un-linked fluorescence molecules.

After establishing the acute pneumonia model for 4 h, free cy5.5, cy5.5@Lip, cy5.5@cryo-leukocyte and cy5.5@Lip/cryo-leukocyte were intravenously injected into mice. The mice were euthanized and dissected at 0.5, 1, 2 and 4 h post-injection. The fluorescence intensities of the organs (heart, liver, spleen, lung and kidney) were imaged using an IVIS imaging system. Quantification of the average radiant efficiency in the organs was analyzed by Living Image software and the radiance percentage of each organ was calculated *via* division by total fluorescence signal of all organs.

The percentage were calculated by the following equation:


$$\mathrm{Percentage}=\frac{F_{\mathrm{tissue}}}{F_{\mathrm{total}}}\times 100\%$$

in which F_tissue_ is the fluorescence intensities of indicated tissue and F_total_ is the sum of the fluorescence intensities of all the tissues.

In a separate animal batch of the pneumonia model, free drugs (MP and BAI), drug@cryo-leukocyte, drug@Lip and drug@Lip/cryo-leukocyte were intravenously injected. Two hours later, the mice were euthanized and typical organs of heart, liver, spleen, lung and kidney tissues were isolated. After washing with saline and dried with filter paper, the tissues were homogenated, and the drug concentration in each tissue sample was quantified with HPLC.

### *In vivo* anti-inflammation efficacy

C57BL/6J mice were anesthetized with isoflurane and 50 μL LPS (10 mg/kg) was instilled into the trachea *via* endotracheal intubation to build up the pneumonia model. The mice were randomly assigned to the groups of saline, MP (6 mg/kg), MP&BAI (MP 6 mg/kg, BAI 5 mg/kg), drug@Lip (MP 6 mg/kg, BAI 5 mg/kg) and drug@Lip/cryo-leukocyte (2 × 10^6^ cryo-leukocyte, MP 6 mg/kg, BAI 5 mg/kg). After model set-up, above formulations were intravenously administrated. 24 h later, peripheral blood was first collected *via* the orbital vein and the cytokine levels in the serum were measured by ELISA kits. After blood collection, the mice were sacrificed and bronchoalveolar lavage fluid was collected. In brief, a 22 G catheter was inserted into the trachea and 1 mL cold PBS (containing 100 μM EDTA) was slowly injected into the lungs for lavage. The collection of lavage fluid was repeated three times. After centrifugation (800 g, 10 min), the protein level in the bronchoalveolar lavage fluid was evaluated by BCA protein detection kit. The precipitated cells were resuspended and incubated with 200 μL ACK buffer for 2 min to remove erythrocytes. After centrifugation at 400 *g* for 7 min, the remaining cells were resuspended in PBS (1% FBS) and the percentages of macrophage (F4/80^+^CD11b^+^) and neutrophil (Ly6G^+^CD11b^+^) were analyzed by flow cytometry after antibody staining. The lung tissues were scissored out for following assays. The right lung lobe was paraffin-embedded and sliced for H&E staining. The left lung lobe was weighed as the wet weight, and the dry weight was recorded after drying at 65°C for 72 h. The wet/dry weight ratio of the lung tissue was calculated to evaluate the extent of pulmonary edema. To evaluate the survival of mice, the procedures of model set-up and treatments were same as stated above, and the mice survival was recorded in a separate animal batch.

### Statistical analysis

The results from at least three independent experiments were presented as mean ± standard deviation (SD). The difference between the data of any two groups was calculated by the two-tailed Student’s *t*-test. The statistical significance was indicated as *P < 0.05; **P < 0.01; ***P < 0.001; ****P < 0.0001; n.s., no significance. All statistical analyses were performed by GraphPad Prism Software.

## Results

### Preparation and biocompatibility of cryo-leukocytes

**Figure 2:**
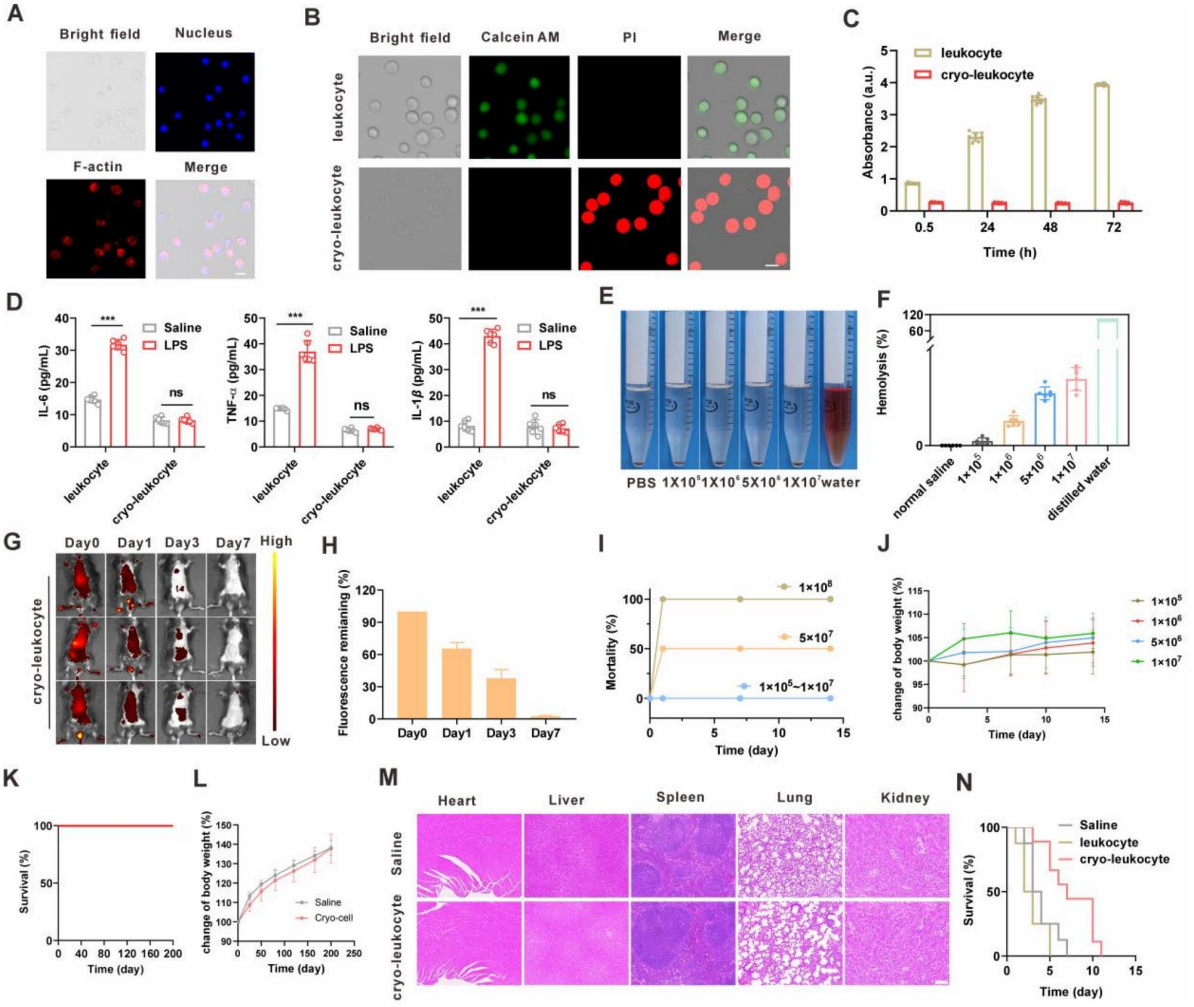
Preparation and characterization of cryo-leukocytes. (**A**) Confocal images of cryo-leukocytes. The nucleus was stained with Hoechst 33342 and the cytoplasmic F-actin was stained with rhodamine phalloidin. Scale bar, 20 μm. (**B**) The cell state of the normal leukocyte and cryo-leukocyte. Calcein AM: live cells; PI: dead cells. Scale bar, 20 μm. (**C**) Cell proliferation analyzed by CCK-8 assay (*n *= 8). (**D**) Cytokine secretion of normal- and cryo-leukocytes after LPS stimulation (*n *= 6). (**E**) Typical images of erythrocyte hemolysis after incubation with cryo-leukocyte. (**F**) The percentage of hemolysis of erythrocytes after incubation with cryo-leukocytes at 37°C for 1 h (*n *= 6). Complete hemolysis occurred in the group of distilled water, which was defined as 100%. (**G**) Typical *in vivo* fluorescence images of mice treated with cy5.5@cryo-leukocyte. (**H**) Statistical analysis of fluorescence remaining of cy5.5@cryo-leukocyte within 7 day after administration (*n *= 3). (**I**) The mortality of mice in acute toxicity test (*n *= 10). (**J**) The body mass of mice in acute toxicity test (*n *= 10). (**K**) Survival of the healthy mice after intravenous injection of 5 × 10^6^ cryo-leukocyte (*n *= 5). (**L**) Body weight of mice in indicated groups during observation period of 200 days (*n *= 5). (**M**) Tissue H&E staining images of the cryo-leukocyte-treated mice. Scale bar, 100 μm. (**N**) Survival of pneumonia model mice after injection of 5 × 10^6^ normal- or cryo-leukocyte (*n *= 8). Data are presented as mean ± SD and the statistical analysis was performed *via* two-tailed Student’s *t*-test between two groups and ordinary one-way ANOVA for three or more groups. ***P < 0.001.

The cryo-leukocytes were obtained *via* the quick shock of peripheral leukocytes in liquid nitrogen and the cellular structure was visualized by CLSM after fluorescence staining of nucleus and F-actin. The cryo-leukocytes preserved relatively intact cellular structure (Figure 2A), but without cell viability (Figure 2B) and proliferation (Figure 2C). In addition, the cryo-leukocytes lost the capability to secrete cytokines. As shown in Figure 2D, after stimulation by LPS, the cytokine levels in the cell culture medium of normal leukocytes were significantly elevated, but no significant difference was observed for cryo-leukocytes.

The biocompatibility of cryo-leukocyte was further evaluated. For blood compatibility, there was seldom hemolysis of the erythrocytes after incubating with cryo-leukocytes and the hemolysis rate was only 2.2% when the cell density of cryo-leukocytes was high up to 5 × 10^6^ cells/mL (Figure 2E and F), far lower than the general standard of well blood compatibility of 5%. After intravenous injection, cryo-leukocytes underwent *in vivo* clearance gradually and the fluorescence signal of cryo-leukocyte was hardly remaining after 7 days (Figure 2G and H).

Furthermore, we tested the acute toxicity of cryo-leukocytes. There were no obvious adverse effects of the mice administered with cryo-leukocytes (1 × 10^5^ – 1 × 10^7^), as evidenced by the evaluation of mice survival, body weight and H&E staining images (Figure 2I and J, Figure S1). However, if the cell amount reached up to 5 × 10^7^ or 1 × 10^8^, the mortality of mice was 50% and 100%, respectively, indicating the safe dose of cryo-leukocyte was 1 × 10^7^ per mouse.

Moreover, we tested the long-term biocompatibility of cryo-leukocyte. As shown in Figure 2K and L, all mice administered with cryo-leukocyte (5 × 10^6^ cells) survived within the observation period of 200 days, with no obvious adverse effects or significant difference in body weight between the cryo-leukocyte-treated mice and healthy control. Typical H&E staining images of primary organs (heart, liver, spleen, lungs and kidneys) also revealed no obvious inflammation appeared (Figure 2M). The biosafety of cryo-leukocyte was also evaluated in LPS-induced pneumonia mice, as shown in Figure 2N, compared with saline (non-treated) and normal leukocyte group, the administration of cryo-leukocyte would not shorten but prolong the survival of pneumonia mice.

### Leveraging cryo-leukocyte as cytokine absorption material

**Figure 3:**
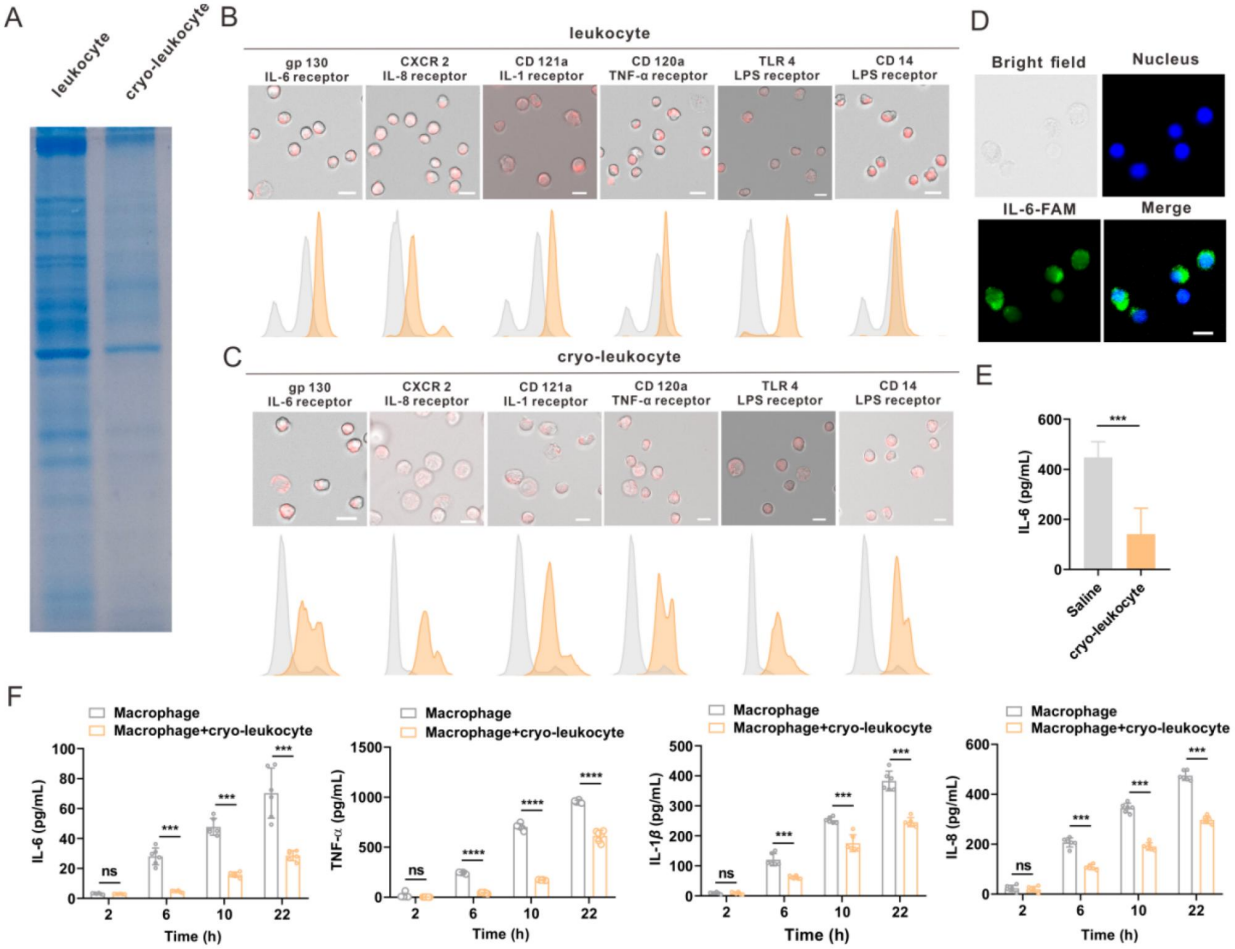
(**A**) SDS-PAGE image of whole-cell lysate proteins obtained from leukocytes and cryo-leukocytes. The cytokine and LPS receptors in leukocyte (**B**) and cryo-leukocyte (**C**) as analyzed by confocal microscopy (top) and flow cytometry (bottom) (scale bar, 20 μm). (**D**) Confocal images of cryo-leukocytes after incubating with FAM-labeled IL-6. Scale bar, 20 μm. (**E**) Changes of IL-6 concentration detected by ELISA kit after incubation with cryo-leukocyte (*n *= 5). (**F**) Cytokine secretion by macrophages at different time points after LPS stimulation (*n *= 6). Data are presented as mean ± SD and the statistical analysis was performed *via* two-tailed Student’s *t*-test between two groups and ordinary one-way ANOVA for three or more groups. ***P < 0.001, ****P < 0.0001.

The total protein expression of cryo-leukocyte was first analyzed by SDS-PAGE and proved to be similar to that of normal leukocytes (Figure 3A). In specific, important acute pneumonia and immune activation-related receptors of IL-6, IL-1β and TNF-α were reserved in cryo-leukocytes (Figure 3B and C). The capability of cryo-leukocyte to adhesion cytokines was further proved. After incubation with fluorescence-labeled IL-6, a clear fluorescence signal was observed within cryo-leukocytes and the IL-6 concentration in the medium was also significantly decreased (Figure 3D and E). In addition, cryo-leukocytes were added to the cell culture medium of macrophages. After LPS activation, the concentration of secreted cytokines (IL-6, TNF-α, IL-1β, IL-8) by macrophages at each time point were significantly reduced (Figure 3F). This phenomenon may be attributed to the ‘trapped’ exogenous LPS by LPS receptors existing on cryo-leukocyte, diminishing the stimulation intensity (Figure 3C), as well as the absorption of cytokines that were secreted by macrophages.

### Immunosuppression of different cryo-cells

**Figure 4:**
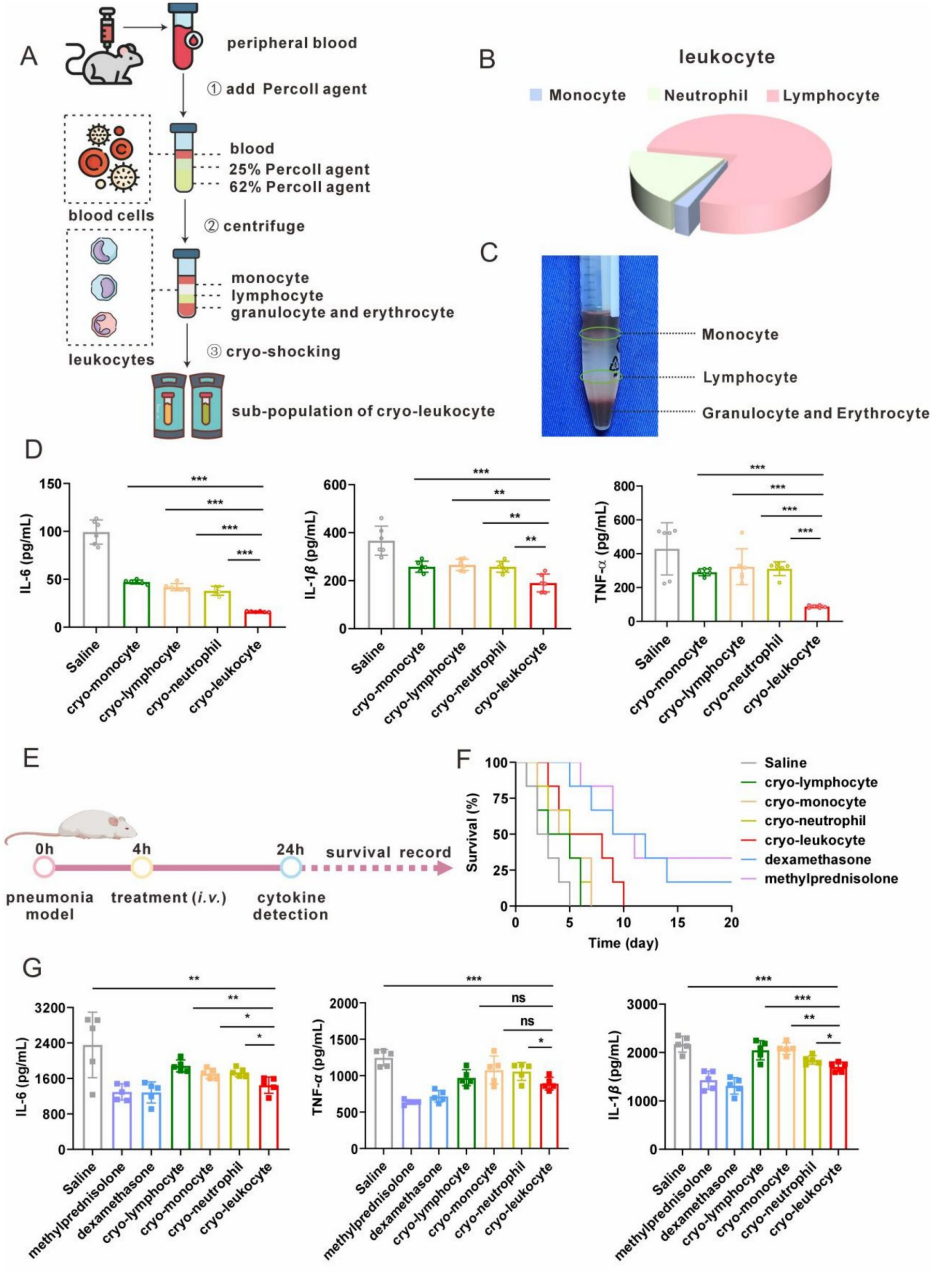
(**A**) Scheme of the separation of leukocytes with density gradient centrifugation and the preparation of cryo-leukocyte subsets. (**B**) Blood routine analysis of C57BL/6J mice (*n *= 6). (**C**) Separation of leukocyte subsets by density gradient centrifugation with Percoll reagent. (**D**) Secreted cytokines by J774A.1 cells after LPS stimulation along with the incubation with different cryo-cells (*n *= 6). (**E**) Scheme of LPS-induced pneumonia model and treatments. (**F**) Survival of LPS-induced pneumonia mice treated with different cryo-cells (*n *= 8). Negative control: saline; positive control: methylprednisolone, dexamethasone. (**G**) Serum cytokine levels of mice after injection of different cryo-cells (5 × 10^6^ cells) (*n *= 6). Negative control: saline; positive control: methylprednisolone, dexamethasone. Data are presented as mean ± SD and the statistical analysis was performed *via* two-tailed Student’s *t*-test between two groups and ordinary one-way ANOVA for three or more groups. *P < 0.05, **P < 0.01, ***P < 0.001.

The leukocytes were sub-grouped by density gradient centrifugation with Percoll reagent (Figure 4A) and the primary compositions of leukocytes in C57BL/6J mice are monocytes (81.5%), granulocytes (7.0%) and lymphocytes (2.4%), as analyzed by automatic blood cell analyzer (Figure 4B). The cell layers after centrifugation were monocytes, lymphocytes and granulocytes from top to bottom (Figure 4C). After preparation of different cryo-cells, the *in vitro* and *in vivo* anti-inflammation efficacy of these cryo-cells were evaluated. All types of cryo-cells exhibited some anti-inflammation effects, as revealed by the decreased cytokines secreted by macrophages after LPS stimulation if incubation with these cryo-cells. However, cryo-leukocyte exhibited stronger anti-inflammation efficacy compared to other single cryo-cells (Figure 4D). As evidenced in acute pneumonia mice model (Figure 4E), after injection of different cryo-cells, the serum cytokine levels showed a significant reduction (Figure 4G), and the median survival of mice were 2 days (saline), 3 days (cryo-lymphocyte), 4 days (cryo-monocyte), 4 days (cryo-neutrophil) and 7 days (cryo-leukocyte), respectively (Figure 4F).

### Proteomic analysis of different cryo-cells

**Figure 5:**
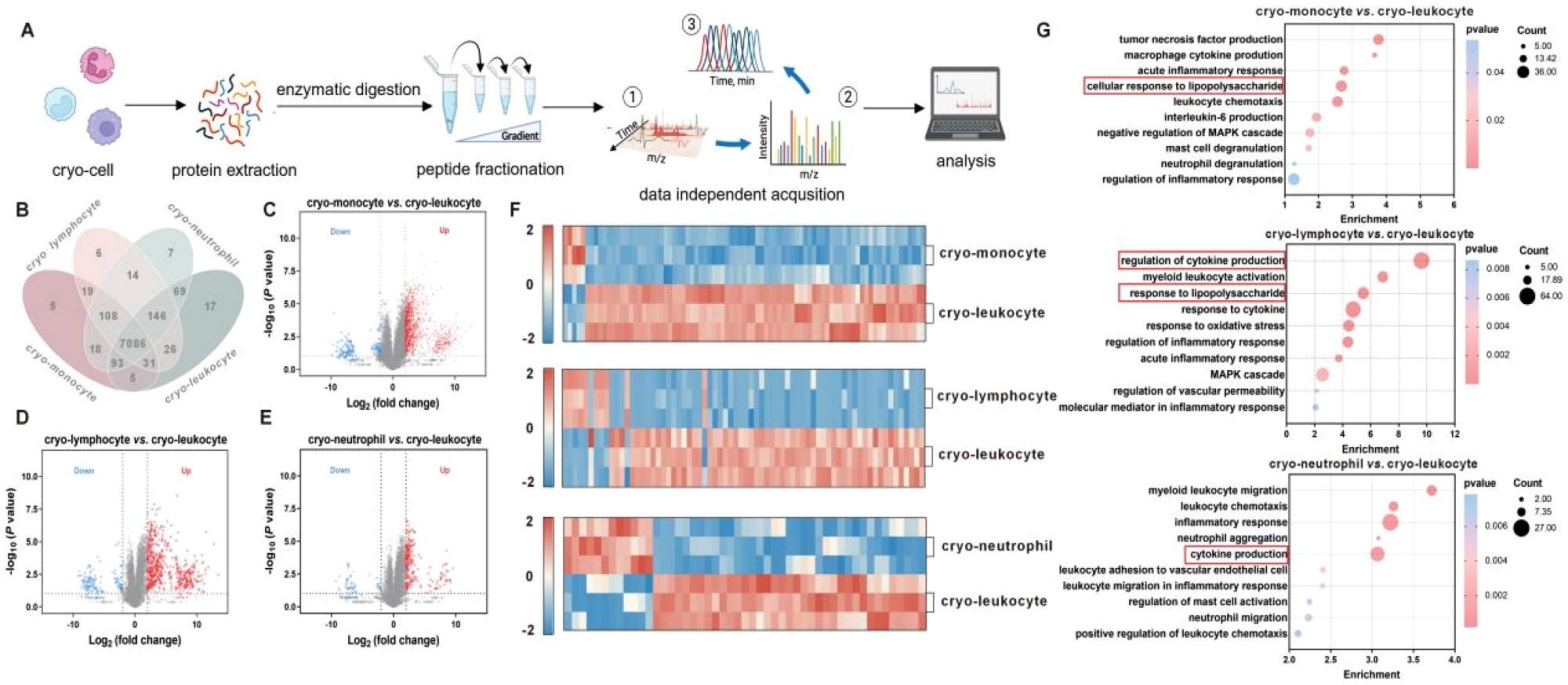
Proteomic analysis of cryo-cell populations. (**A**) Flow chart of the process of proteomic analysis. (**B**) Venn analysis of differentially expressed proteins of the denoted cryo-cells (*n *= 3). (**C**–**E**) Volcano plot of the distribution of differentially expressed proteins in cryo-leukocyte compared with cryo-monocyte, cryo-lymphocyte and cryo-neutrophil. Right: up-regulated proteins; left: down-regulated proteins (*n *= 3). (**F**) Heatmap of relative abundance of differentially expressed proteins between cryo-leukocyte and sub-cryo-leukocyte populations (*n *= 3). (**G**) Potential inflammation-associated biological processes analyzed by GO enrichment in cryo-leukocyte compared with other cryo-cells.

Proteomic analysis was adopted to evaluate the proteins of cryo-cells (Figure 5A). Venn analysis, volcano analysis, hierarchical clustering analysis and GO annotation enrichment analysis were utilized to analyze the differentially expressed proteins between different cryo-cells and the potential inflammation-associated biological processes engaged by these DEPs. The protein expressions of different cryo-cells were almost similar, as about 7086 proteins co-existed in these cells, accounting for 92.6% of all proteins (Figure 5B). However, the protein contents were significantly differentiated between cryo-leukocytes and other cryo-cells. As shown in Figure 5C–F, the number of up-regulated proteins of cryo-leukocyte were 1232, 542 and 209, respectively, compared with cryo-monocyte, cryo-lymphocyte and cryo-neutrophil, while only a quantity of 153, 134 and 55 for down-regulated proteins, respectively.

These up-regulated DEPs mostly participated in regulation of the immune responses (Figure 5G), such as response to LPS, leukocyte activation and cytokine production, etc. Thus, the administration of cryo-leukocyte could behave as the counterpart and camouflage of normal leukocytes to alleviate the inflammation cascades in the body, and exhibited higher anti-inflammation efficacy compared with cryo-monocyte, cryo-lymphocyte and cryo-neutrophil.

### Leveraging cryo-leukocyte and aICAM-1-modified liposomes to construct lung-targeting vehicle

For the treatment of acute pneumonia, the development of lung-targeting system of glucocorticoids can significantly reduce the systemic distribution of drugs, improving drug efficacy and safety. The lung-targeting vehicle in this work was composed of two parts, cryo-leukocyte and aICAM-1-modified liposome. Cryo-leukocyte was to mediate transit lung passive targeting due to its cellular size and aICAM-1-modified liposome was to mediate long-term lung accumulation *via* the anchor effects between ICAM-1 antibody and ICAM-1 molecules highly expressed on inflamed lung vascular endothelial cells.

MP is a synthetic glucocorticoid that can inhibit the inflammatory cascade. Because of its strong anti-inflammatory effects, MP is widely used in the treatment of a variety of acute and chronic inflammatory diseases. Thus, we first chose MP as the therapeutic drug for the treatment of pneumonia. However, large dose and repeated administration of MP will lead to drug resistance and serious local and systemic side effects, such as osteoporosis and femoral head necrosis.

BAI is one of the main flavonoid extracts extracted from *Scutellaria baicalensis*, which is widely used in the combination therapy of acute pneumonia in clinic in China. Thus, we choose the combination of BAI and MP to achieve the synergistic anti-inflammation effects.

We explored the combination index (CI) of MP and BAI, and verified the synergistic therapeutic effects based on the calculation of CI:


$$Q_{1}/Q_{X1}+Q_{2}/Q_{X2}+\ldots +Q_{n}/Q_{\text{xn}}=\text{CI}$$

As shown in Figure S4 and Table S2, the addition of a small dose of BAI could significantly enhance the anti-inflammation efficacy of MP.

**Figure 6:**
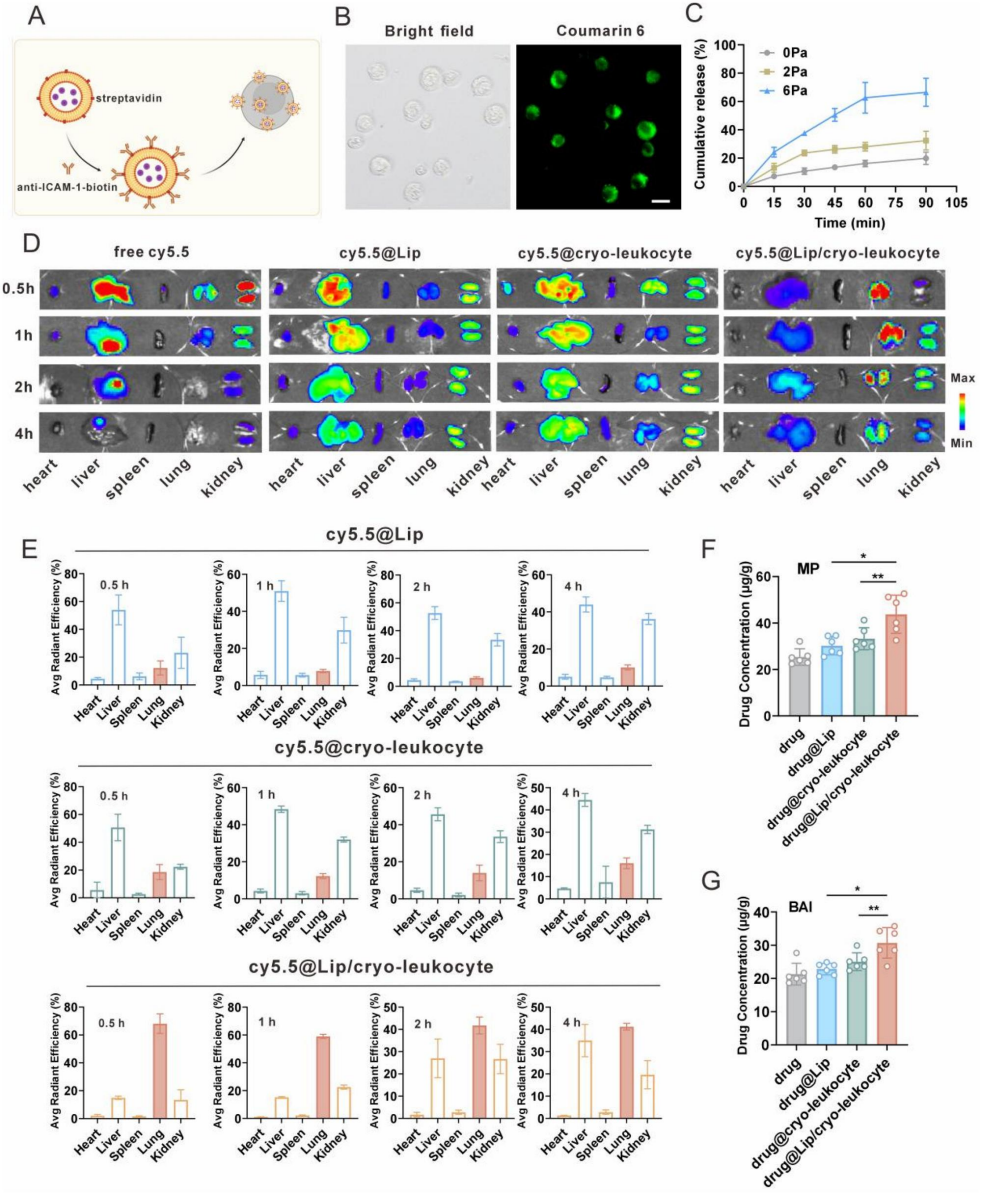
(**A**) Scheme of the preparation of drug-loaded cryo-leukocytes. (**B**) Typical confocal images of drug@Lip/cryo-leukocyte (scale bar, 20 μm). The fluorescence dye of coumarin 6 was adopted as the model drug. (**C**) Drug release from drug@Lip/cryo-leukocyte under different shearing forces (*n *= 5). (**D**) Typical IVIS images of *in vivo* distribution of indicated formulations. (**E**) Percentage of fluorescence radiance of major organs at indicated time points (*n *= 3). (**F** and **G**) Methylprednisolone and baicalin contents in lung tissue of mice after 2 h of *i.v*. administration of indicated formulation (*n *= 6). Data are presented as mean ± SD and the statistical analysis was performed *via* two-tailed Student’s *t*-test between two groups and ordinary one-way ANOVA for three or more groups. *P < 0.05, **P < 0.01, ***P < 0.001.

The anti-inflammation drugs were first loaded in aICAM-1-modified liposomes (Table S1, Figures S2 and S3) with the antibody amount of 9.96 ng per 10 µg liposome and further incubation with negatively charged cryo-leukocytes to obtain drug-loaded cryo-leukocyte (drug@Lip/cryo-leukocyte) (Figure 6A). For characterization of drug@Lip/cryo-leukocyte, coumarin 6 was used as a model drug. As shown in Figure 6B, there was an obvious fluorescence signal in cryo-leukocyte, confirming the successful loading of drugs. The drug-loading capacity of MP and BAI in 1 × 10^6^ cryo-leukocytes was 89.5 ± 4.3 and 74.5 ± 10.2 μg, respectively. The mass ratio of the two drugs was 1.2:1, which was in the synergistic combination range (Figure S4, Table S2). To assess the main factor that influenced drug loading, the drug-loading capacity of liposomes (with or without anti-ICAM-1 modification) on cryo-leukocyte were tested. As shown in Figure S5, the two kinds of liposomes had no significant difference in the drug-loading capacity, 83.6 ± 5.3 vs. 85.3 ± 8.1 μg per 1 × 10^6^ cryo-leukocyte, respectively, indicating the electrostatic interaction is the main factor.

The drug release behavior was further assessed with a peristaltic pump to mimic the shear stress of blood flow. According to the literature, the shear stress in pulmonary capillaries is approximately 6 Pa, while in normal blood flow, it is around 2 Pa [25]. As shown in Figure 6C, the drug release could reach 62.6% at the shear stress of 6 Pa within 1 h, while only 16.2% at 0 Pa and 28.1% at 2 Pa, indicating the fast detachment of drug-loaded liposomes from cryo-leukocyte in pulmonary capillaries. Then, the drugs could be further released from liposomes in a sustained manner (Figures S6 and S7).

We further analyzed the lung-targeting efficacy of drug@Lip/cryo-leukocyte. The fluorescence dye of cy5.5 was adopted as the model drug to visualize the distribution of the formulations *in vivo*. As shown in Figure 6D, free cy5.5 was rapidly cleared post-injection and mostly distributed in liver and kidney. In contrast, the clearance of cy5.5 in other groups was reduced, indicating that the *in vivo* half-life of small molecules could be extended using liposomes or cryo-leukocytes as drug vehicles, but tissue distribution varied among the groups. The nano-sized cy5.5@Lip mostly distributed in liver (>50%), while a small amount in lung with the fraction of 12.2% (0.5 h), 7.8% (1 h), 6.0% (2 h) (Figure 5E and Figure S8). The cryo-leukocytes were approximately 10 μm in size and had a high deformation rate, their distribution in lung after intravenous injection was 18.6% (0.5 h), but decreased gradually, indicating transit and limited retention of cryo-leukocyte in lungs (Figure S8). In contrast, the delivery system combining drug@Lip and cryo-leukocyte enabled long-term drug accumulation in lungs, and the fraction of cy5.5@Lip/cryo-leukocyte distributed in lungs was 68.1% (0.5 h), 59.0% (1 h), 41.7% (2 h) and 41.2% (4 h) (Figure 6E and Figure S9), significantly higher than other groups. Besides, we also evaluated the lung retention behavior between the two groups of anti-ICAM-1-Lip/cryo-cell and Lip/cryo-cell in a separated batch of animals with lung distribution percentages of 65.8 vs. 22.6% (0.5 h) and 63.3 vs. 14.2% (1 h), respectively (Figure S9).

We detected the drug contents of MP and BAI in lungs of different groups by tissue distribution test. As shown in Figure 6F, both MP and BAI in the drug@Lip/cryo-leukocyte group were significantly higher than the other groups (Figure 6F).

### *In vivo* anti-inflammation efficacy

**Figure 7:**
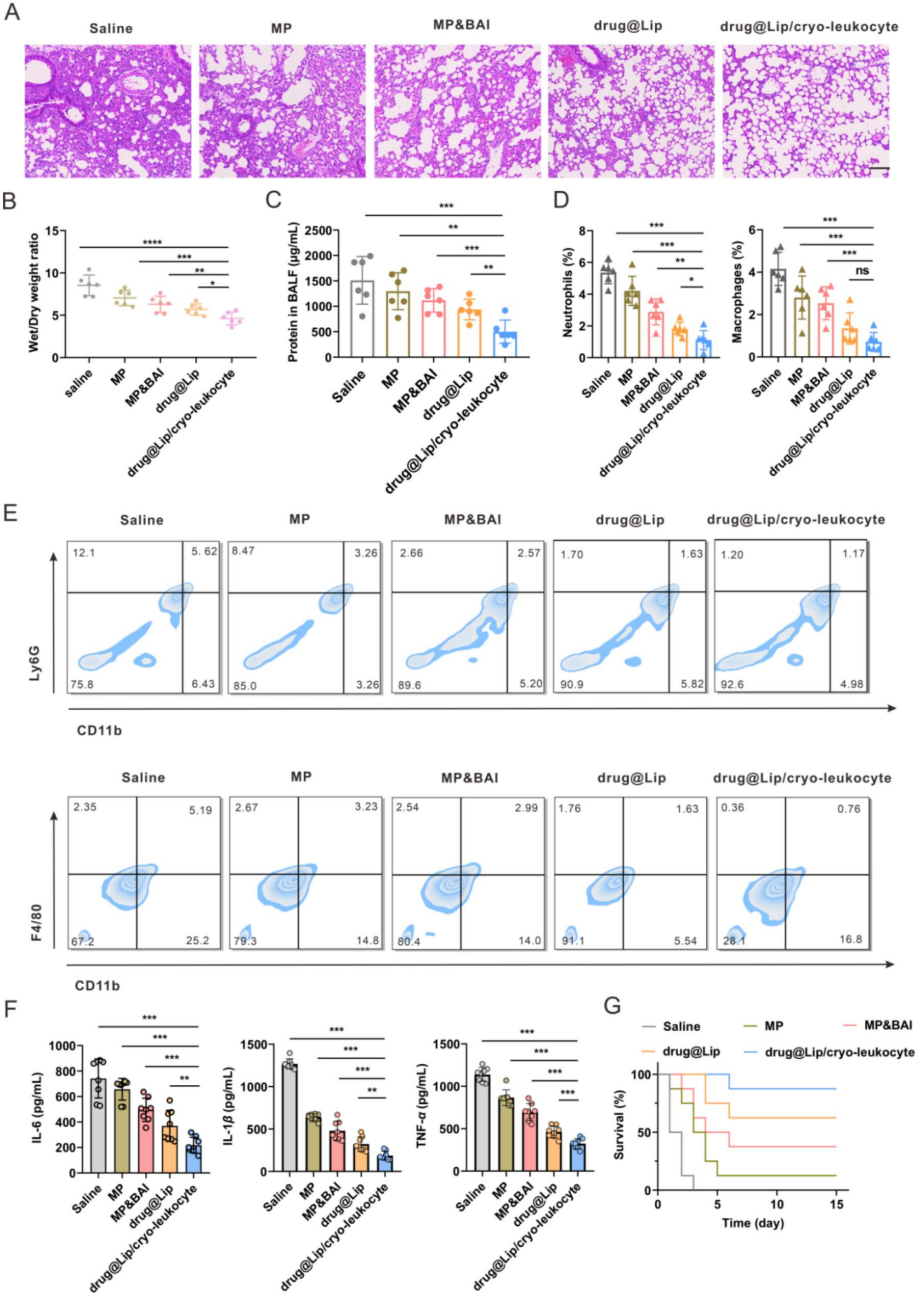
*In vivo* anti-inflammation efficacy of drug@lip/cryo-leukocyte in LPS-induced acute pneumonia model. (**A**) Typical images of lung tissues after H&E staining in different groups (scale bar, 100 μm). (**B**) Wet/dry weight ratio of lung tissue (*n *= 6). (**C**) Total protein level in BAL fluid (*n *= 6). (**D**) Flow cytometry percentages of neutrophils (left) and macrophages (right) in BAL fluid (*n *= 6). (**E**) Typical flow cytometry images of neutrophils (top) and macrophages (bottom) in BAL fluid. (**F**) Serum cytokine levels of IL-6, IL-1β and TNF-α in different groups (*n *= 8). (**G**) Survival of the pneumonia mice in different treatment groups (*n *= 8). Data are presented as mean ± SD and the statistical analysis was performed *via* two-tailed Student’s *t*-test between two groups and ordinary one-way ANOVA for three or more groups. *P < 0.05, **P < 0.01, ***P < 0.001.

LPS was intratracheally instilled into the mice to set up the pneumonia model. After treatment with saline, MP, MP&BAI, drug@Lip and drug@Lip/cryo-leukocyte, the inflammation state of the lung was evaluated. As shown in Figure 7A, the extent of lung injury in the drug@Lip/cryo-leukocyte group was the most modest compared to the other groups, with thinner alveolar walls, lower wet/dry weight ratio of the lung (indication of pulmonary edema) (Figure 7B), smaller protein concentration in bronchoalveolar lavage fluid (indication of pulmonary capillary permeability) (Figure 7C) and less infiltration of both monocyte-derived macrophages (F4/80^+^ CD11b^+^) and neutrophils (Ly6G^+^ CD11b^+^) (Figure 7D and E).

In addition, the serum cytokine levels were also detected. Compared with the untreated group, the cytokine levels including IL-6, IL-1β and TNF-α were significantly decreased after treatment with drug@Lip/cryo-leukocyte (Figure 7F). In specific, the serum IL-6 concentrations, an important indicator of acute pneumonia, were 1213 and 512 pg/mL, respectively for the saline and formulation-treated groups. And for normal mice (without LPS stimulation and no treatments), serum IL-6 concentration was 126 pg/mL, indicating the well anti-inflammation efficacy of drug@Lip/cryo-leukocyte [25]. In addition, the drug efficacy was better than that of free drug and drug@Lip, achieving 87.5% survival in the pneumonia mice model (Figure 7G).

## Discussion

Biofunctional and biomimetic materials play vital role in disease treatments [26–38]. In this work, we evaluated the efficacy, safety and feasibility of cryo-leukocyte as the cytokine absorption biomaterial and drug delivery vehicle for anti-inflammation treatment.

Regarding efficacy, in contrast to conventional single-antibody neutralization therapy, cryo-leukocyte could behave as ‘mixed’ antibodies by binding various cytokines such as IL-6, IL-1, IL-8 and TNF-α, thus exhibit well anti-inflammation efficacy as evidenced by comparative studies with standard glucocorticoid treatments of MP or dexamethasone in LPS-induced pneumonia model (Figure 4E and F). Moreover, severe inflammation usually represents a cascade amplification of the immune system [39], involving dozens of immune cells, thus individual cryo-immune cell types (e.g. single population) may exhibit inferior immunomodulatory potency compared to the whole cryo-leukocytes. Our preliminary studies isolated monocytes, neutrophils and lymphocytes from whole blood, followed by comparative evaluation of their anti-inflammation efficacy versus cryo-leukocytes both *in vitro* and *in vivo*. The results consistently demonstrated that cryo-leukocyte possessed enhanced immunosuppression capacity (Figure 4C, E and F).

Regarding biosafety, leukocytes are autologously sourced, exhibiting low immunogenicity. Administration of appropriate doses of cryo-leukocyte did not induce significant toxic side effects. In this work, we conducted comprehensive biocompatibility assessments of the cryo-leukocytes, including immunogenicity (Figure 2D), hematotoxicity (Figure 2F), acute toxicity, long-term toxicity and the evaluation of safety dose thresholds (Figure 2I–M, Figure S1). These investigations provide preliminary validation of the clinical safety of cryo-leukocytes.

Regarding feasibility, the preparation of cryo-leukocyte requires five streamlined steps: whole blood collection, erythrocyte depletion, cryo-shocking by liquid nitrogen, thawing/washing and patient reinfusion. This simplified workflow enables bed-to-bed therapeutic implementation, demonstrating exceptional clinical feasibility.

Cryo-leukocyte serve as lung-targeting vehicle and exert anti-inflammatory efficacy, which exhibited advantages to other cell-based systems to some extent. For example, the cryo-leukocyte could exhibit better anti-inflammation capability than erythrocyte-based or other membrane-based systems.

Lung-targeting drug delivery system has unique advantages in the treatment of pulmonary diseases [40–43], and cryo-leukocyte represented a naturally passive but transit lung-targeting capacity due to its cellular size [44], and after coupling with aICAM-1 modified drug-loaded liposomes can achieve obvious anchoring effect in inflammation sites and long retention of drugs in lung tissues. Compared to conventional polymer-based carriers, these cell-derived micro-sized vehicles exhibit well deformability and could leave lung capillaries along with blood flow, eliminating the risk of vascular occlusion, and drug loading could be achieved by electrostatic interaction *via* mixing drug-loaded cationic liposomes with cryo-leukocytes, which is of well practical feasibility.

## Conclusion

Cryo-leukocyte-based anti-inflammation biologics was constructed, in which cryo-leukocyte behaved as the cytokine absorption material, and its efficacy was superior than that of its sub-group cells of cryo-neutrophil, cryo-monocyte and cryo-lymphocyte. The micro/nano composite system together with cryo-leukocyte and aICAM-1 functionalized liposome could achieve obvious drug lung-targeting delivery and exhibit synergetic anti-inflammation efficacy along with the loaded drugs to alleviate pulmonary inflammation.

## Supplementary Material

rbag032_Supplementary_Data

## Data Availability

Data will be made available on request.
