# Supplementary material for: Biomimetic lung-targeting vehicle coupled with cryo-shocked leukocyte and inflammation-anchored liposome for drug delivery and anti-inflammation in treatment of acute pneumonia
Source: Regen Biomater. 2026 Mar 10;13:rbag032. doi: 10.1093/rb/rbag032 (PMC13198373; doi:10.1093/rb/rbag032)
Supplement: rbag032_Supplementary_Data [file rbag032_supplementary_data.docx]

Supplementary Information

Biomimetic lung-targeting vehicle coupled with cryo-shocked leukocyte and inflammation-anchored liposome for drug delivery and anti-inflammation in treatment of acute pneumonia

**This PDF file includes:**

Figure S1. H&E staining images of typical organs of mice after administration of cryo-leukocytes (scale bar, 100 μm).

Figure S2. TEM image of drug-loaded liposomes (scale bar, 50 μm).

Figure S3. Size change of drug@aICAM-Lip during storage in PBS at 4℃ (*n* = 3).

Figure S4. Screening of the combination doses of methylprednisolone and baicalin.

Figure S5. The loading capacity of coumarin 6 per 1×10^6^ cryo-cell (*n* = 3).

Figure S6**.** *In vitro* release of methylprednisolone and baicalin from drug-loaded liposomes (*n* = 3).

Figure S7. *In vitro* release of coumarin 6 from coumarin 6-loaded liposomes (*n* = 3).

Figure S8. Percentage of fluorescence radiance of lungs at indicated time points.

Figure S9. (A) Typical IVIS images of *in vivo* distribution of cy5.5@Lip/cryo-leukocyte and cy5.5@aICAM-Lip/cryo-leukocyte. (B) Percentage of fluorescence radiance of major organs at indicated time points.

Table S1. Physical and chemical properties of drug-loaded liposomes.

Table S2. Combination index of methylprednisolone and baicalin.

Materials and Methods

Screening of MP and BAI combination proportions

The synergistic anti-inflammatory effects of methylprednisolone (MP) and baicalin (BAI) were assessed by evaluating the cytokine secretion of J774A.1 cells after stimulating with lipopolysaccharide (LPS). J774.1A macrophages were seeded in 24-well plates, LPS (50 ng/mL) was added, and MP solution (5, 15, 30, 60 μM) or BAI solution (3, 6, 30, 60 μM) was added 2 h later. After further incubation for 6 h, the concentration of IL-6 in the supernatant fluid of each well was measured by ELISA kit. The half-maximal inhibitory concentration MP (IC_50, MP_) and BAI (IC_50, BAI_) were calculated using CompuSyn software 1.2 software.

The anti-inflammatory effects of MP and BAI in different combination proportions were evaluated, and the combination index (CI) was calculated to determine whether there was a synergistic effect between the two drugs. J774.1A cells were seeded into 24-well plates, and LPS (50 ng/mL) was added to each well to stimulate J774.1A cells for 2 h. The combination ratios of MP and BAI were set as follows: 100% IC_50, MP_, 80% IC_50, MP_ + 20% IC_50, BAI_, 70% IC_50, MP_ + 30% IC_50, BAI_, 60% IC_50, MP_ + 40% IC_50, BAI_, 50% IC_50, MP_ + 50% IC_50, BAI_, 40% IC_50, MP_ + 60% IC_50, BAI_, 30% IC_50, MP_ + 70% IC_50, BAI_, 20% IC_50, MP_ + 80% IC_50, BAI_ and 100% IC_50, BAI_. The drugs with different proportions were added to each well, and incubate for another 6 h, the concentrations of IL-6 in the supernatant culture medium of the different groups were measured by ELISA kit and CI values of MP and BAI at different combination ratios were calculated by Compusyn 1.2 software. CI ＜1: synergistic effect; CI =1: additive effect; CI ＞1: antagonistic effect.

Characterization of drug-loaded liposomes

The particle size, polydispersity index (PDI) and zeta potential of drug-loaded liposomes were determined by dynamic light scattering (DLS, Nazo ZS 90, UK Malvern Inc.). The encapsulation efficiency (EE) and drug loading capacity (DL) were determined by HPLC according to standard determination protocol. After negatively stained by uranyl acetate, the morphology of liposomes was observed by transmission electron microscope (TEM, JEM-2100F, JEOL Japan).

*In vitro* drug release of drug@Lip

2.4 mL of drug@Lip was added in the dialysis bag (Mw 3500 Da), with 80 mL PBS as the releasing medium. At 10 min, 20 min, 30 min, 1 h, 2 h, 4 h, 8 h, and 12 h, 2 mL PBS was withdrawn and the concentrations of MP and BAI in the solution were determined by HPLC. The fluorescence model drug release from cy5.5@Lip and coumarin 6@Lip were determined with the same method by microplate reader.

Stability of drug-loaded liposomes

The liposome solution was stored at 4°C for 25 days, and the samples were taken out at day 1, day 7, day 14 and day 25. After dilution with PBS, the particle size was measured by DLS.

Lung targeting of drug@Lip/cryo-leukocyte

Free cy5.5 was used as the model drug and encapsulated in anti-ICAM-1 modified liposomes to prepare cy5.5@aICAM-Lip. The positively charged cy5.5@aICAM-Lip was then incubated with cryo-leukocyte to obtain cy5.5@aICAM-Lip/cryo-leukocyte. Also, cy5.5-loaded liposome without anti-ICAM-1 was incubated with cryo-leukocyte to obtain cy5.5@Lip/cryo-leukocyte. The fluorescence signal of heart, liver, spleen, lung, kidney for each experimental group at every time point was recorded, and the percentage was calculated.


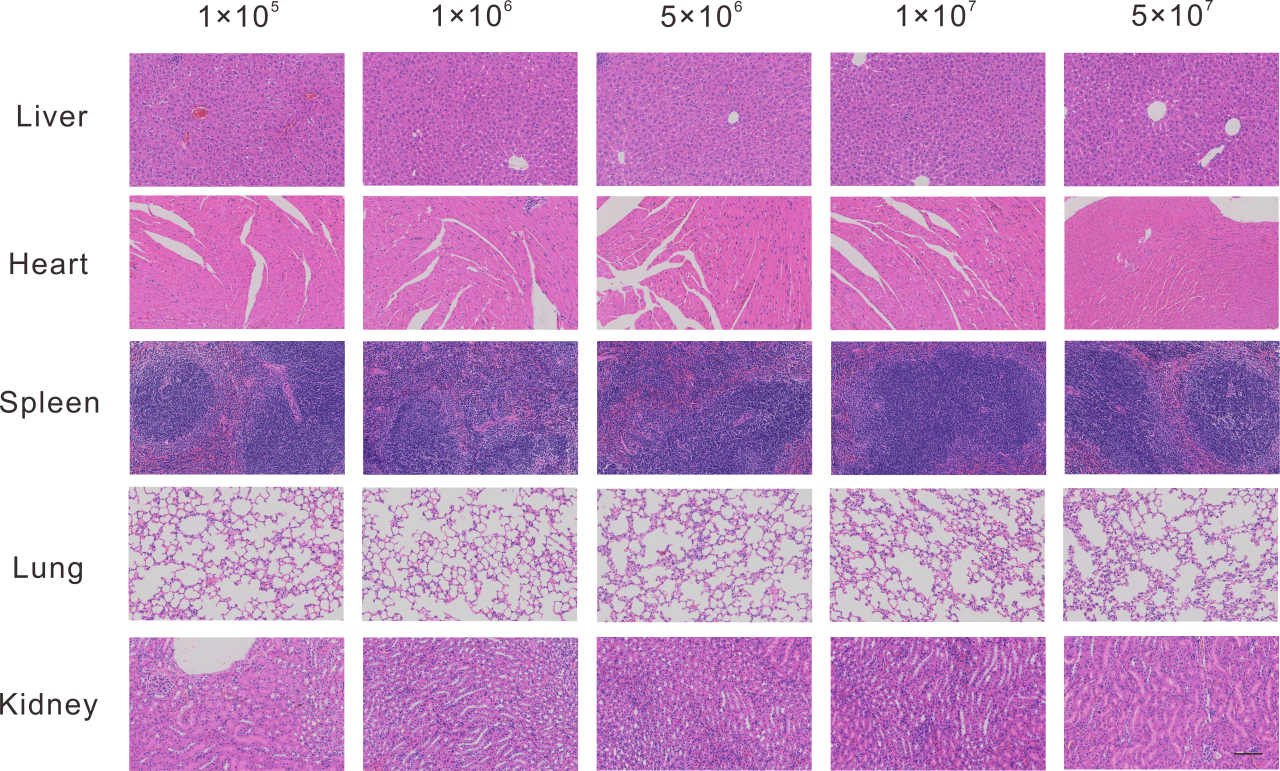


**Figure S1.** H&E staining images of typical organs of mice after administration of cryo-leukocytes (scale bar, 100 μm).


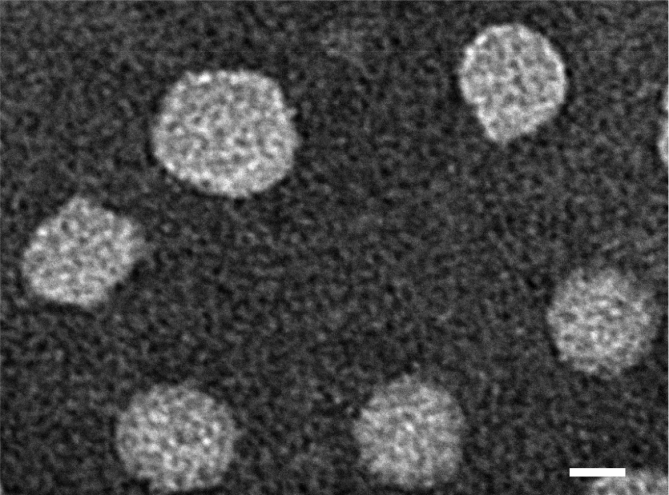


**Figure S2.** TEM image of drug-loaded liposomes (scale bar, 50 μm).


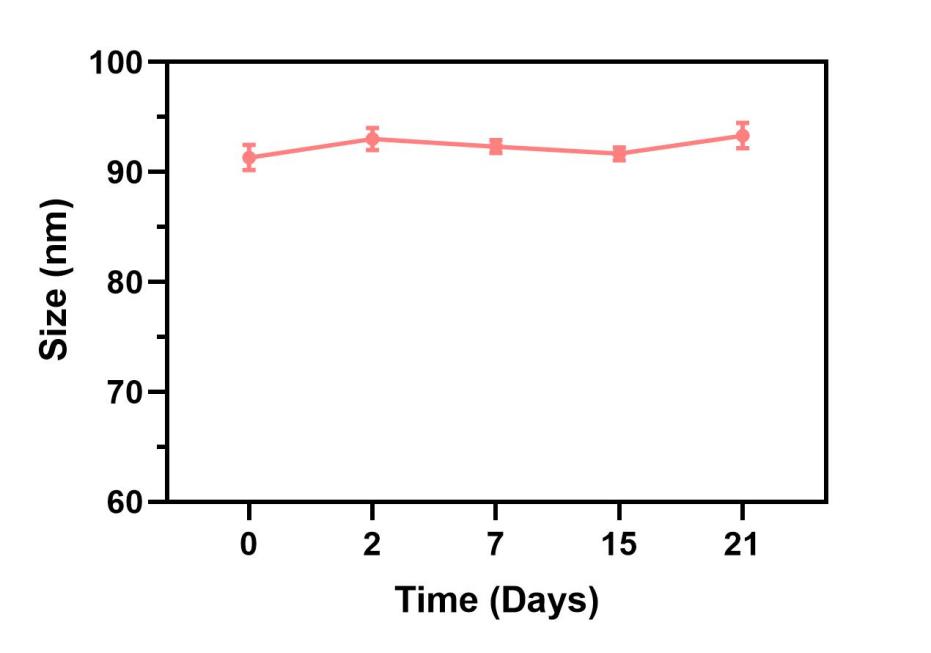


**Figure S3**. Size change of drug@aICAM-Lip during storage in PBS at 4℃ (*n* = 3).


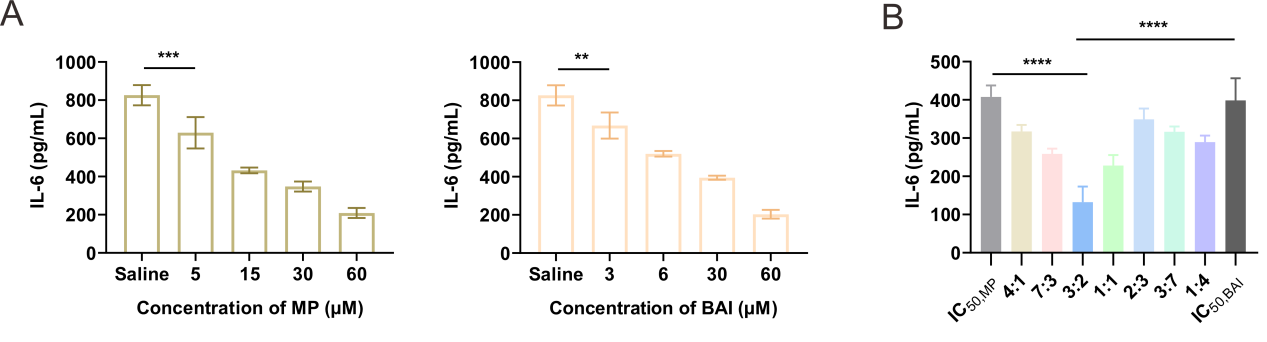


**Figure S4.** Screening of the combination doses of methylprednisolone and baicalin. (A) IL-6 secretion of J774A.1 cells activated by LPS after treatment with methylprednisolone (left) or baicalin (right) (*n* = 6). (B) IL-6 secretion of J774A.1 cells after treatment with indicated combination proportions of MP and BAI (*n* = 6). The combination ratios of MP and BAI were set as follows: 100% IC_50, MP_, 80% IC_50, MP_ + 20% IC_50, BAI_, 70% IC_50, MP_ + 30% IC_50, BAI_, 60% IC_50, MP_ + 40% IC_50, BAI_, 50% IC_50, MP_ + 50% IC_50, BAI_, 40% IC_50, MP_ + 60% IC_50, BAI_, 30% IC_50, MP_ + 70% IC_50, BAI_, 20% IC_50, MP_ + 80% IC_50, BAI_ and 100% IC_50, BAI_. Data are presented as mean ± SD. Statistical significance was calculated *via* Student’s *t*-test. ***P* < 0.01, ****P* < 0.001.


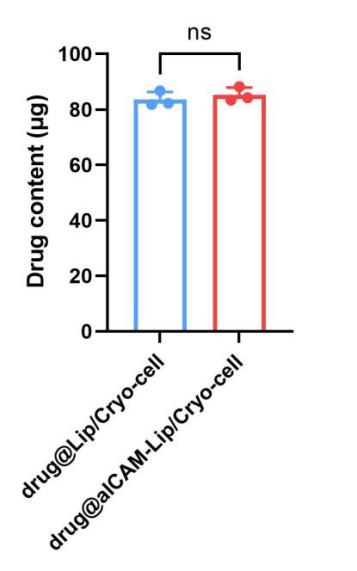


**Figure S5.** The loading capacity of coumarin 6 per 1×10^6^ cryo-cell (*n* = 3).


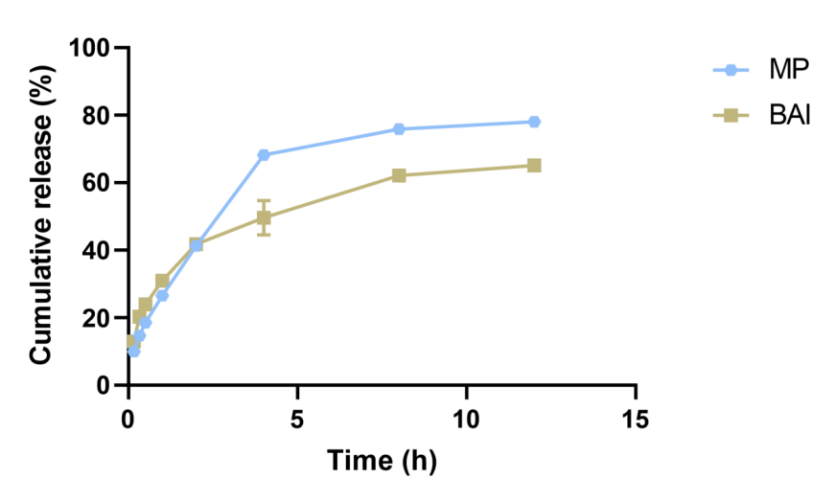


**Figure S6**. *In* *vitro* release of methylprednisolone and baicalin from drug-loaded liposomes (n = 3).


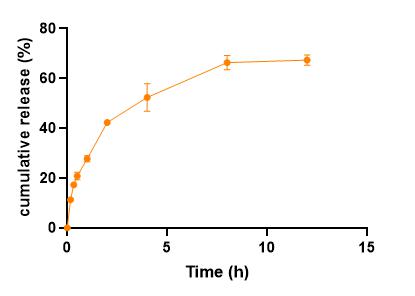


**Figure S7**. *In vitro* release of coumarin 6 from coumarin 6-loaded liposomes (*n* = 3).


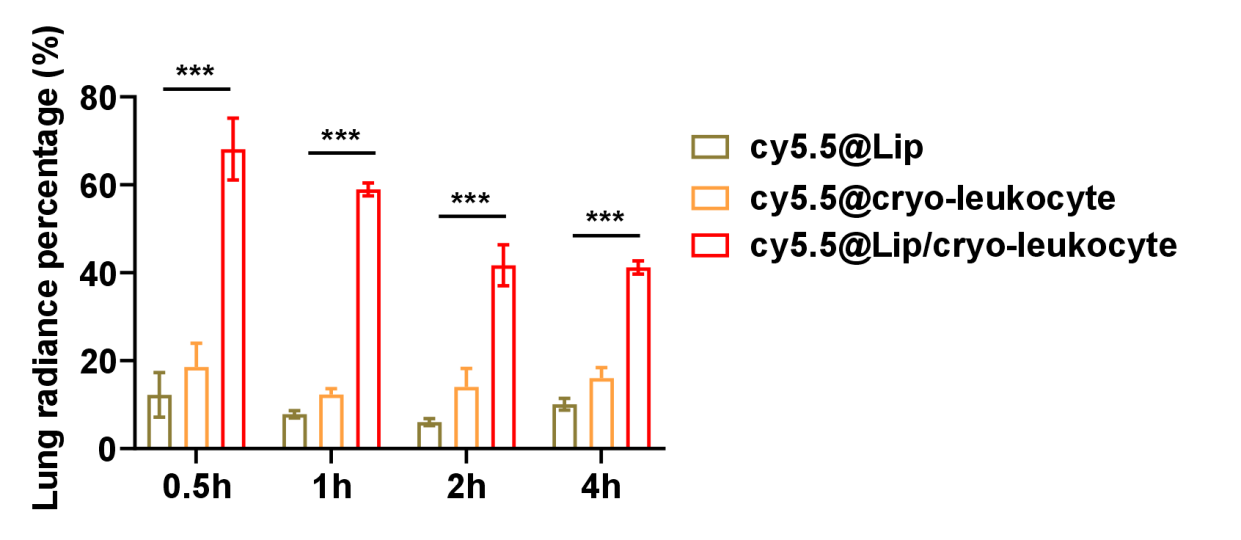


**Figure S8.** Percentage of fluorescence radiance of lungs at indicated time points. Data are presented as mean ± SD (*n* = 3).


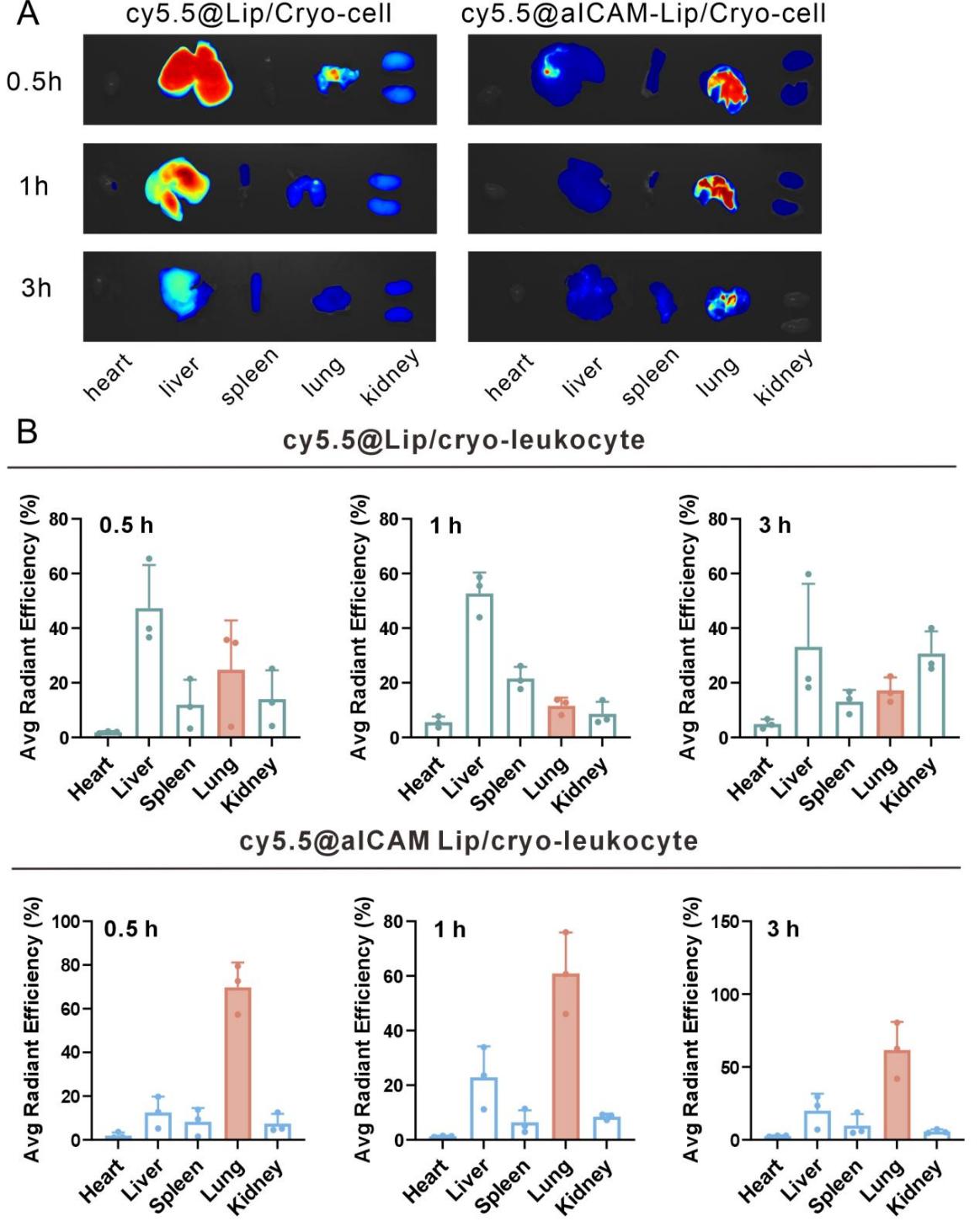


**Figure S9**. (A) Typical IVIS images of *in vivo* distribution of cy5.5@Lip/cryo-leukocyte and cy5.5@aICAM-Lip/cryo-leukocyte. (B) Percentage of fluorescence radiance of major organs at indicated time points.

**Table S1.** Physical and chemical properties of drug-loaded liposomes (*n* = 3).

| Size (nm) | PDI | Zeta (mV) | EE (%) | | DL (%) | |
| --- | --- | --- | --- | --- | --- | --- |
| 82.50±2.67 | 0.224±0.03 | 14.53±1.57 | MP | BAI | MP | BAI |
|  |  |  | 79.10±4.74 | 79.71±4.90 | 5.31±0.89 | 5.44±1.13 |

**Table S2.** Combination index of methylprednisolone and baicalin.

| Drug | Concentration  of MP [μM] | Concentration  of BAI [μM] | CI |
| --- | --- | --- | --- |
| IC_50, MP_ | 19.05 |  |  |
| 80% IC_50, MP_ : 20% IC_50, BAI_ | 15.24 | 4.24 | 0.63 |
| 70% IC_50, MP_ : 30% IC_50, BAI_ | 13.34 | 6.36 | 0.74 |
| 60% IC_50, MP_ : 40% IC_50, BAI_ | 11.43 | 8.48 | 0.57 |
| 50% IC_50, MP_ : 50% IC_50, BAI_ | 9.53 | 10.6 | 0.72 |
| 40% IC_50, MP_ : 60% IC_50, BAI_ | 7.62 | 12.72 | 0.85 |
| 30% IC_50, MP_ :70% IC_50, BAI_ | 5.72 | 14.84 | 0.61 |
| 20% IC_50, MP_ : 80% IC_50, BAI_ | 3.81 | 16.96 | 0.65 |
| IC_50, BAI_ |  | 21.20 |  |
